# Additions to the Inventory of the Genus *Alternaria* Section *Alternaria* (*Pleosporaceae*, *Pleosporales*) in Italy

**DOI:** 10.3390/jof8090898

**Published:** 2022-08-24

**Authors:** Junfu Li, Rungtiwa Phookamsak, Hongbo Jiang, Darbhe Jayarama Bhat, Erio Camporesi, Saisamorn Lumyong, Jaturong Kumla, Sinang Hongsanan, Peter E. Mortimer, Jianchu Xu, Nakarin Suwannarach

**Affiliations:** 1 Honghe Center for Mountain Futures, Kunming Institute of Botany, Chinese Academy of Sciences, Honghe 654400, China; 2 Department of Economic Plants and Biotechnology, Yunnan Key Laboratory for Wild Plant Resources, Kunming Institute of Botany, Chinese Academy of Sciences, Kunming 650201, China; 3 East and Central Asia Regional Office, World Agroforestry Centre (ICRAF), Kunming 650201, China; 4 Centre for Mountain Futures (CMF), Kunming Institute of Botany, Kunming 650201, China; 5 Research Center of Microbial Diversity and Sustainable Utilization, Faculty of Sciences, Chiang Mai University, Chiang Mai 50200, Thailand; 6 No. 128/1-J, Azad Housing Society, Curca, P.O. Box, Goa Velha 403108, India; 7 Società per gli Studi Naturalistici della Romagna, C.P. 143, 48012 Bagnacavallo, RA, Italy; 8 Department of Biology, Faculty of Science, Chiang Mai University, Chiang Mai 50200, Thailand; 9 Academy of Science, The Royal Society of Thailand, Bangkok 10300, Thailand; 10 Shenzhen Key Laboratory of Microbial Genetic Engineering, College of Life Sciences and Oceanography, Shenzhen University, Shenzhen 518060, China; 11 Department of Entomology and Plant Pathology, Faculty of Agriculture, Chiang Mai University, Chiang Mai 50200, Thailand

**Keywords:** *Dothideomycetes*, Italian dematiaceous hyphomycetes, multi-locus phylogeny, saprobic fungi, taxonomy

## Abstract

The genus *Alternaria* is comprised of well-known plant pathogens causing various important diseases in plants, as well as being common allergens in animals and humans. Species of *Alternaria* can be found as saprobes associated with various dead plant materials. This research aims to enhance the taxonomy of saprobic species in the genus *Alternaria* found on grasses and herbaceous plants from Italy, based on multi-locus phylogenetic analyses of a concatenated ITS, LSU, SSU, *tef1-α*, *rpb2*, *gapdh* and *Alt-a1* DNA sequence dataset combined with morphological characteristics. Multi-locus phylogenetic analyses demonstrated six novel species belonging to the genus *Alternaria* sect. *Alternaria* as: *A. muriformispora* sp. nov., *A. obpyriconidia* sp. nov., *A. ovoidea* sp. nov., *A. pseudoinfectoria* sp. nov., *A. rostroconidia* sp. nov. and *A. torilis* sp. nov. Detailed morphological descriptions, illustrations and an updated phylogenetic relationship of taxa in the genus *Alternaria* sect. *Alternaria* are provided herein.

## 1. Introduction

The genus *Alternaria* is classified in the family *Pleosporaceae*, order *Pleosporales*, class *Dothideomycetes* [1,2,3]. The genus contains over 700 species epithets [4], but approximately 378 species are accepted within 28 sections, of which less than 100 species have molecular data to clarify their phylogenetic affinities [1,2,3,5,6,7]. Species of *Alternaria* are well-known as serious plant pathogens and post-harvest pathogens, causing major crop losses, or can be the causative agents of animal and human pathogens, causing phaeohyphomycosis or acting as airborne allergens [8,9,10,11].

*Alternaria* is well-known as dematiaceous hyphomycetes which can be found everywhere. The genus is characterized by mononematous, macro- or micronematous, unbranched or branched conidiophores, integrated to discrete, mono- to polytretic conidiogenous cells, solitary or catenate, straight or curved, phragmo- or dictyoseptate, smooth or verrucose and median brown to dark brown conidia with rounded or narrowly-beaked tip. *Alternaria* occupies diverse ecological niches through its life modes, which range from endophytes to pathogens to saprobes on a wide range of host substrates (e.g., agricultural products, animals, plants, seeds, soil as well as the atmosphere) [2,8,10,11,12]. The genus has a cosmopolitan distribution, and is widely distributed in Asia, Australia, Europe, and North America [13]. 

Lawrence et al. [14] introduced *Alternaria* sect. *Alternaria* to accommodate *Alternaria* species, commonly referred to small-spored *Alternaria* groups. The members of *Alternaria* sect. *Alternaria* can be morphologically distinguished from other sections in having small conidia produced in short chains (frequently less than 60 µm in length *in vitro*) [8,14,15]. However, this small-spored criterion is not significant to distinguish species in *Alternaria* sect. *Alternaria* from other *Alternaria* sections, when multi-locus phylogeny has become an essential tool to discriminate species in *Alternaria* [2]. According to Li et al. [2], some species in *Alternaria* sect. *Alternaria* have conidia larger than 60 µm, but these species were affiliated with *Alternaria* sect. *Alternaria* based on multi-locus phylogenetic evidence. The holomorph of sect. *Alternaria* is known for *A. alternata*, the generic type of the section, and the sexual morph is described as typically erumpent, small-sized, smooth, globose to ovoid, dark brown; with papillate ascomata; cylindrical to cylindric-clavate asci and muriform, ellipsoid to fusoid, brown, eguttulate, smooth-walled ascospores [2,10,16]. Woudenberg et al. [8] estimated 60 species accommodating in sect. *Alternaria* based on ITS gene analysis. Consequently, Woudenberg et al. [17] accepted only 11 species and one species complex in this section based on polyphasic taxonomic approaches, while 35 morphospecies were treated as synonyms of *A. alternata*. Later, Li et al. [2] showed that these 35 synonymized species can be divided into 5 main subclades in their analyses of *A. alternata*, pending questions on their conspecific status. Gannibal [15] re-circumscribed and amended the section based on morphological assessments by Simmons [18], and included the other 37 morphospecies and accepted 59 species in sect. *Alternaria*. Subsequently, the other four species (i.e., *A. calystegiae*, *A. diversispora*, *A. guaranitica* and *A. macalpinei*) were included in this section by Gannibal and Lawrence [19]. *Alternaria doliconidium* and *A. italica* were also included in this section by Wanasinghe et al. [20] and Jayawardena et al. [21] respectively. Nishikawa and Nakashima [22] also included *A. iridicola* in this section. Recently, Li et al. [2] introduced another 14 species in sect. *Alternaria.* Therefore, 83 species are currently accommodated in this section.

Recent molecular phylogenetic studies have shown that the identification of species in *Alternaria* and its close relative genera challenged their morphological basis [8,14,17,23,24,25,26,27,28]. In general, the molecular data tends to support the recent morphologically distinct sub-generic species groups [8,10,14,29]. However, the phylogenetic relationships of the *Alternaria* sections are normally variable, with the morphological characteristics used to identify morphospecies. On the other hand, Woudenberg et al. [8] delineate species in *Alternaria* sect. *Alternaria* based on ITS. The whole-genome sequencing has become an essential tool to delineate ambiguous species in *Alternaria* and other complex species by Woudenberg et al. [17]. Thus, Woudenberg et al. [17] used multi-locus phylogeny based on ITS, *gapdh*, *rpb2*, *tef1-α*, *Alt-a1*, *endoPG* and *OPA10-2* gene loci coupled with whole-genome and transcriptome comparisons to discriminate species in sect. *Alternaria*, and accepted only 11 phylogenetic species and one species complex in *Alternaria* sect. *Alternaria*. Furthermore, Woudenberg et al. [17] synonymized 35 morphospecies under *A. alternata*. In addition, the lack of phylogenetic effective coding genes led to confusion in the identification of *Alternaria* species [8,10,17]; therefore, re-defining and expanding the generic concept of *Alternaria* sect. *Alternaria* and other *Alternaria* sections is necessary. These studies suggest that morphological characteristics typically used to delineate species (e.g., conidium length, width and septation; chain structure; and beak shape) may not reflect evolutionary relationships between taxa.

*Alternaria* species are major plant pathogens that infect a vast array of plant hosts [2,8,10,11,15,30]. Members in *Alternaria* sect. *Alternaria* are still confused in their delineation of species which are largely based on morphology and the clarity of their host species. The present study aims to introduce six novel species in *Alternaria* sect. *Alternaria* on different specific plant hosts based on a morpho-molecular approach.

## 2. Materials and Methods

### 2.1. Collection, Examination, Isolation, and Conservation

Samples were collected from dead branches, stems, and twigs of several plant hosts in Italy. The samples were dried and preserved in paper bags for further observation and examination under an Olympus SZ61 series stereo microscope. Micro-morphological features were mounted in sterilized distilled water on a clean slide for examination, and captured by a Nikon DS-Ri2 camera under a Nikon ECLIPSE Ni compound microscope. The size of micro-morphological features was measured by using Tarosoft (R) Image FrameWork version 0.9.7. Photographic plates were edited and combined in Adobe Photoshop CS6 software (Adobe Systems Inc., San Jose, CA, USA). The type specimens were deposited at the herbarium of Mae Fah Luang University, Chiang Rai, Thailand (MFLU).

Axenic cultures were obtained from single spore isolation using a spore suspension technique described by Senanayake et al. [31]. Germinated conidia were aseptically cultivated on potato dextrose agar (PDA) or malt extract agar (MEA) media under day/night lighting at room temperature (25–30 °C). The growth of fungal colonies and sporulation in cultures were observed after two weeks and eight weeks of incubation, respectively. The ex-type living cultures were deposited in the Mae Fah Luang University Culture Collection (MFLUCC). The novel species were registered in Index Fungorum (http://www.indexfungorum.org/names/IndexFungorumRegister.htm, accessed on 15 July 2022). 

### 2.2. DNA Extraction, PCR Amplification, and Sequencing

Fungal genomic DNA were extracted from fresh mycelia growing on PDA/MEA for one month using the Biospin Fungus Genomic DNA Extraction Kit (BioFlux^®^, Hangzhou, China). The duplicated strain of each species was extracted DNA from fungal fruiting bodies using Forensic DNA Kit (Omega^®^, Norcross, GA, USA).

DNA fragments were amplified by polymerase chain reaction (PCR) with seven gene loci, including the internal transcribed spacers (ITS: ITS1-5.8S-ITS2) using primers ITS5 and ITS4 [32], the 28S large subunit rDNA (LSU) using primers LR0R and LR5 [33], the 18S small subunit rDNA (SSU) using primers NS1 and NS4 [32], the partial RNA polymerase second largest subunit (*rpb2*) using primers fRPB2-5F and fRPB2-7cR [34], the translation elongation factor 1-alpha (*tef1-α*) using primers EF1-728F and EF1-986R [35], *Alternaria* major allergen (*Alt-a1*) using primers ALT-F and ALT-R [25] and Glyceraldehyde 3-phosphate Dehydrogenase (*gapdh*) using primers GDP-1 and GDP-2 [36]. The polymerase chain reaction (PCR) was performed in a Veriti™ 96-Well Fast Thermal Cycler (Applied Biosystem, California, USA) following the protocol described in Li et al. [2]. All PCR products were sent to TsingKe Biological Technology (Beijing) Co., Ltd., China for purification and sequencing. The quality of the sanger DNA sequences and sequence consensus from forward and reward directions was checked and assembled manually in BioEdit v. 7.2.3 [37], and the newly nucleotide sequences were deposited in GenBank (Table 1).

### 2.3. Sequence Alignment and Phylogenetic Analyses

The newly generated ITS, LSU, SSU, *tef1-α*, *rpb2*, *gapdh* and *Alt*-*a1* sequences were subjected to the nucleotide BLAST search engine via the NCBI (https://www.ncbi.nlm.nih.gov/, accessed on 10 April 2022) for checking potential contaminants or erroneous sequences as well as delineating the closely related taxa. All reference sequences were downloaded from GenBank. The multiple sequence matrixes were automatically aligned by MAFFT v. 7.452 (https://mafft.cbrc.jp/alignment/software/, accessed on 20 May 2022) [38]. Manual improvements were made where necessary in BioEdit v. 7.2.3 [37]. Individual gene alignments were separately analyzed by maximum likelihood (ML) in order to check the congruence of tree topology, and, thus, the combined multi-locus phylogenetic trees were inferred based on Bayesian inference (BI) and maximum likelihood (ML) analyses. 

Maximum likelihood (ML) analyses were performed by Randomized Axelerated Maximum Likelihood (RAxML) [39,40] implemented in raxmlGUI 1.3 [41] using the default setting, but adjusted with 1000 bootstrap replicates and a GAMMAI model of nucleotide substitution. MrModeltest v. 2.3 [42] was used to determine the best-fit model of nucleotide substitution for each locus and incorporated into the analyses. GTR+I+G was the best-fit model for ITS, LSU and *Alt-a1* loci under the Akaike Information Criterion (AIC), while TIM2+I+G was the best-fit model for SSU and *rpb2*, SYM+I+G was the best-fit model for *gapdh* and TIM1+I+G was the best-fit model for *tef1-α*. Bayesian inference (BI) analyses were performed by MrBayes v.3.1.2 [43]. Markov Chain Monte Carlo (MCMC) of six simultaneous Markov chains was run with one million generations to determine posterior probabilities (PP) [44,45], and started from a random tree topology. Trees were frequently sampled at 100th generation and the temperature value of heated chain was set to 0.15. The extra runs were required when the average standard deviation of split frequencies did not lower than 0.01 after one million generation. The first 25% trees represented the burn-in phase of the analyses and were discarded. The remaining trees were used for calculating posterior probabilities (PP) in the majority rule consensus tree. The phylogram were visualized in FigTree v. 1.4.0 [46] and edited in Microsoft Office PowerPoint 2016 (Microsoft Inc., Redmond, WA, USA). 

## 3. Results

### 3.1. Phylogeny

**Figure 1:**
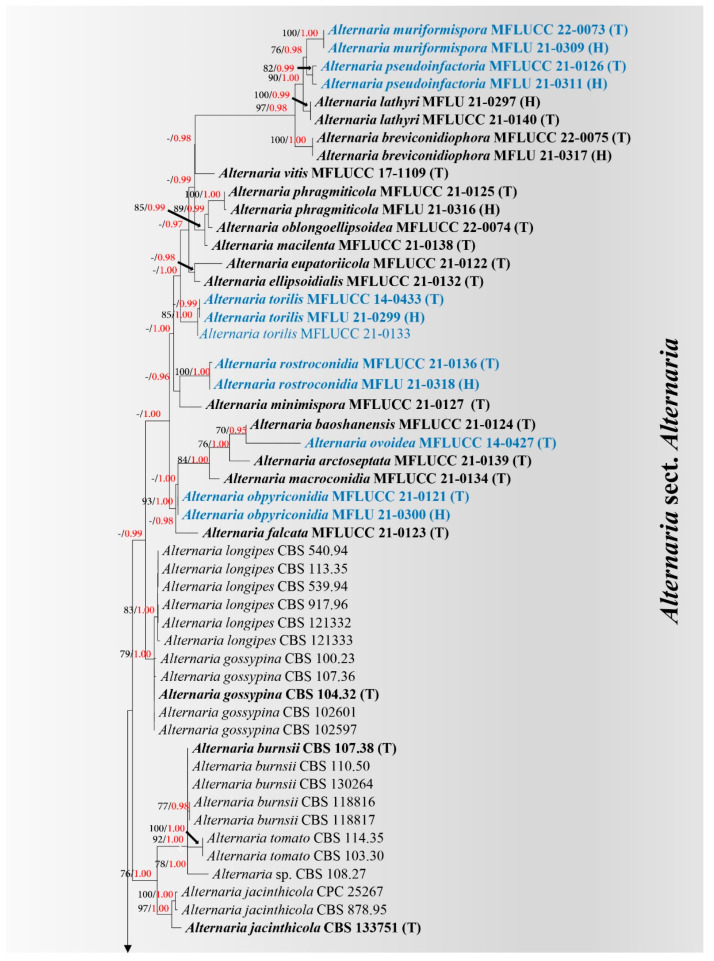

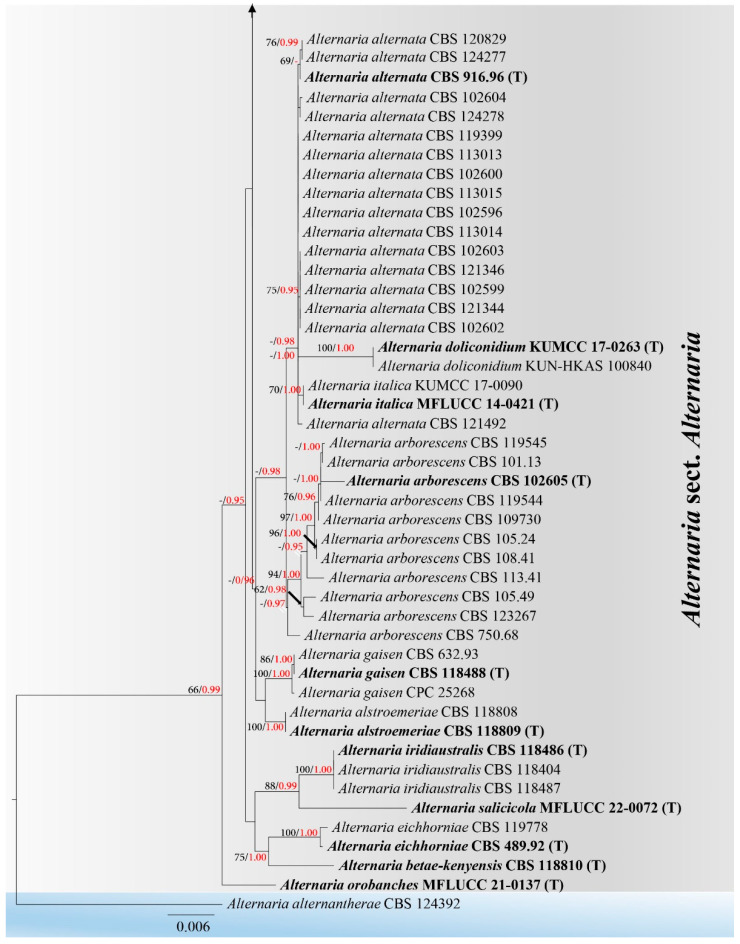
Phylogenetic tree of *Alternaria* sect. *Alternaria* generated by RAxML-based analysis of a combined ITS, LSU, SSU, *tef1-α, rpb2*, *gapdh* and *Alt-a1* DNA sequence dataset. Bootstrap support values for maximum likelihood (ML, black) equal to or greater than 60% and Bayesian posterior probabilities (PP, red) equal to or greater than 0.95 PP are shown above the nodes. The tree is rooted to *Alternaria alternantherae* (CBS 124392). Newly species and generated strains are in blue, and the type strains are indicated in bold. Strains obtained from ex-type living culture are indicated by (T) and strains obtained from holotype specimen are indicated by (H).

Six new species collected from dead herbaceous and monocotyledonous plants in Italy were analyzed with other representative *Alternaria* species in sect. *Alternaria* including *Alternaria muriformispora* (strain MFLUCC 22-0073; on *Plantago* sp.), *A. obpyriconidia* (strains MFLUCC 21-0121 and MFLUCC 14-0435; on *Vicia faba*), *A. ovoidea* (MFLUCC 14-0427; on *Dactylis glomerata*), *A. pseudoinfectoria* (MFLUCC 21-0126; on *Chenopodium* sp.), *A. rostroconidia* (MFLUCC 21-0136; on *Arabis* sp.) and *A. torilis* (MFLUCC 14-0433 and MFLUCC 21-0133; on *Torilis arvensis*). The analyses represented phylogenetic relationships of taxa in *Alternaria* sect. *Alternaria* as well as the placement of six new species. Phylogenetic construction of sect. *Alternaria* based on a combined ITS, LSU, SSU, *tef1-α, rpb2, gapdh* and *Alt-a1* DNA sequence dataset comprises 96 sequences of 34 representative species in sect. *Alternaria*, and *Alternaria alternantherae* (CBS 124392) was selected as the outgroup taxon. The best scoring RAxML tree is shown in Figure 1 with the final ML optimization likelihood value of -11313.333238 (ln). The dataset consists of 4377 total characters, including gaps (ITS: 1–514 bp, LSU: 515–1368 bp, SSU: 1369–2295 bp, *tef1-α*: 2296–2540 bp, *rpb2*: 2541–3311 bp, *gapdh*: 3312–3897 bp, *Alt-a1*: 3898–4377 bp). RAxML analysis yielded 511 distinct alignment patterns and 8.3% of undetermined characters or gaps. Estimated base frequencies were as follows: A = 0.246733, C = 0.254032, G = 0.258489, T = 0.240746, with substitution rates AC = 0.896323, AG = 2.073824, AT = 1.043150, CG = 0.820017, CT = 4.179461 and GT = 1.000000. The gamma distribution shape parameter alpha = 0.020013 and the Tree-Length = 0.230674. Bayesian posterior probabilities (PP) from MCMC were evaluated with a final average standard deviation of split frequencies = 0.008527. 

Multi-locus phylogenetic analyses based on ML and BI criteria showed overall similarity in tree topologies. *Alternaria muriformispora* (MFLUCC 22-0073, MFLU 21-0309) has a close phylogenetic relationship with *A. pseudoinfectoria* (MFLUCC 21-0126, MFLU 21-0311) (76% ML, 0.98 PP; Figure 1) and also clustered with *A. lathyri* (MFLUCC 21-0140, MFLU 21-0297) and *A. breviconidiophora* (MFLUCC 22-0075, MFLU 21-0317). These four species formed a well-resolved subclade in sect. *Alternaria* with 97% ML and 0.98 PP support. *Alternaria obpyriconidia* (MFLUCC 21-0121, MFLU 21-0300) formed a clade with *A.* *macroconidia* (MFLUCC 21-0134), *A.* *arctoseptata* (MFLUCC 21-0139), *A. ovoidea* (MFLUCC 14-0427), *A. baoshanensis* (MFLUCC 21-0124) and *A. falcata* (MFLUCC 21-0123) with 93% ML and 1.00 PP support (Figure 1). While *A. ovoidea* (MFLUCC 14-0427) is sister to *A. baoshanensis* (MFLUCC 21-0124) with significant support (70% ML, 0.95 PP), and is also constituted in this clade. *Alternaria rostroconidia* (MFLUCC 21-0136, MFLU 21-0318) formed a separated branch with *A. minimispora* (MFLUCC 21-0127) with significant support in BI analysis (0.96 PP; Figure 1). *Alternaria* *torilis* (MFLUCC 14-0433, MFLUCC 21-0133, MFLU 21-0299) formed an independent subclade, related to *A.* *ellipsoidialis* (MFLUCC 21-0132) and *A. eupatoriicola* (MFLUCC 21-0122).

### 3.2. Taxonomy

**Figure 2:**
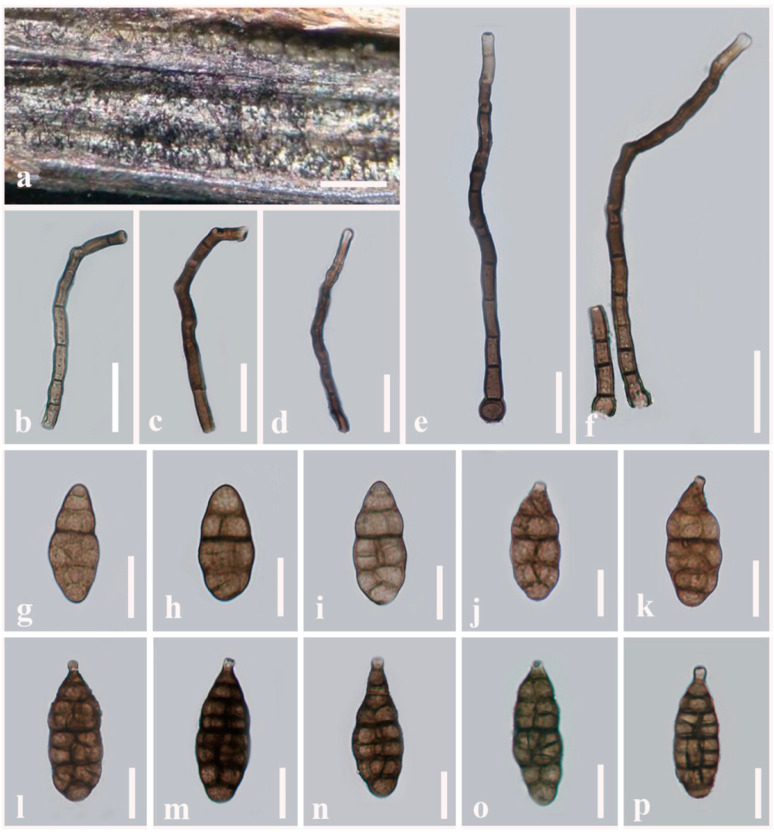
***Alternaria muriformispora*** (MFLU 21-0309, holotype). (**a**) Colonies on dead aerial stem of *Plantago* sp. (*Plantaginaceae*); (**b**–**f**) Conidiophores bearing conidiogenous cells; (**g**–**p**) Conidia. Scale bars: (**a**) = 100 µm, (**b**–**f**) = 50 µm, (**g**,**j**–**p**) = 30 µm, (**h**,**i**) = 20 µm.

***Alternaria muriformispora*** J.F. Li, Camporesi, Phookamsak & Bhat, sp. nov. Figure 2

Index Fungorum number: IF 559795
Etymology: Named after its muriform conidia.
Holotype: MFLU 21-0309

Saprobic on dead aerial stems of *Plantago* sp. (*Plantaginaceae*). Sexual morph: Undetermined. Asexual morph: Mycelium superficial on the substrate, composed of septate, branched, smooth, thin-walled, brown hyphae. Conidiophores 185–201 × 12–13 µm ($\overset{-}{x}$ = 192 × 12 µm, *n* = 30), macronematous, straight or flexuous, cylindrical, with swollen at the basal cell, slightly narrower towards the apex, dark brown, paler at the apex, smooth, septate, unbranched, thick-walled. Conidiogenous cells 4–5 × 5–7 µm ($\overset{-}{x}$ = 4.5 × 6.2 µm, *n* = 20), polytretic, integrated, terminal, determinate or percurrent, cylindrical, subhyaline to light brown, smooth, thin-walled, apically doliiform with one conidiogenous locus. Conidia 75–88 × 23–35 µm ($\overset{-}{x}$ = 83 × 29 µm, *n* = 30), acrogenous, solitary, dry, simple, straight, curved, ellipsoidal to ovoid, or obpyriform with short, narrow, paler brown, aseptate, unbranched, obtuse beak, copper brown to dark brown, four to seven transverse eusepta, with 1–2 longitudinal or oblique or Y-shaped septa in all middle transverse divisions, without oblique or longitudinal septa at both end cells, slightly thickened and constricted at middle septa, borne in chain, verruculose to verrucose, thin-walled. Conidial secession schizolytic.

Culture characteristics: Conidia germinating on PDA within 14 h and germ tubes produced from lateral cells. Colonies hairy or cottony, brown to dark brown, reaching 5 cm in 7 days at 25 °C, mycelium superficial, effuse, radially striate, with irregular edge, brown to dark brown hyphae; conidia not formed *in vitro* within 60 days.

Material examined: Italy, Province of Forlì-Cesena, Meldola, on dead aerial stems of *Plantago* sp. (*Plantaginaceae*), 8 September 2014, E. Camporesi, IT2101 (MFLU 21-0309, holotype), ex-type living culture = MFLUCC 22-0073.

Notes: Multi-locus phylogeny showed that two strains of *Alternaria muriformispora* formed a robust clade (100% ML, 1.00 PP; Figure 1) sister to *A. pseudoinfectoria* with moderate support (76% ML, 0.98 PP; Figure 1). *Alternaria muriformispora* differs from *A. pseudoinfectoria* in having larger (83 × 29 µm vs. 33 × 19 µm), ovoid to ellipsoidal, or obpyriform, short beak and copper brown to dark brown conidia, with 4–7 transverse eusepta and 1–2 longitudinal or oblique or Y-shaped septa in all middle transverse divisions. *Alternaria pseudoinfectoria* has subglobose to obclavate, or obpyriform, light brown conidia, with 3–4 transverse eusepta and 1–2 longitudinal or oblique or Y-shaped septa and conidia that form long, cylindrical, septate, unbranched secondary conidiophores with one apical conidiogenous locus. A nucleotide pairwise comparison of *rpb2* sequences showed that *A. muriformispora* differs from *A. pseudoinfectoria* in 10/559 bp (1.8% difference, no gap). In *Alt-a1*, *A. muriformispora* differs from *A. pseudoinfectoria* in 9/474 bp (1.9% difference, no gap). 

**Figure 3:**
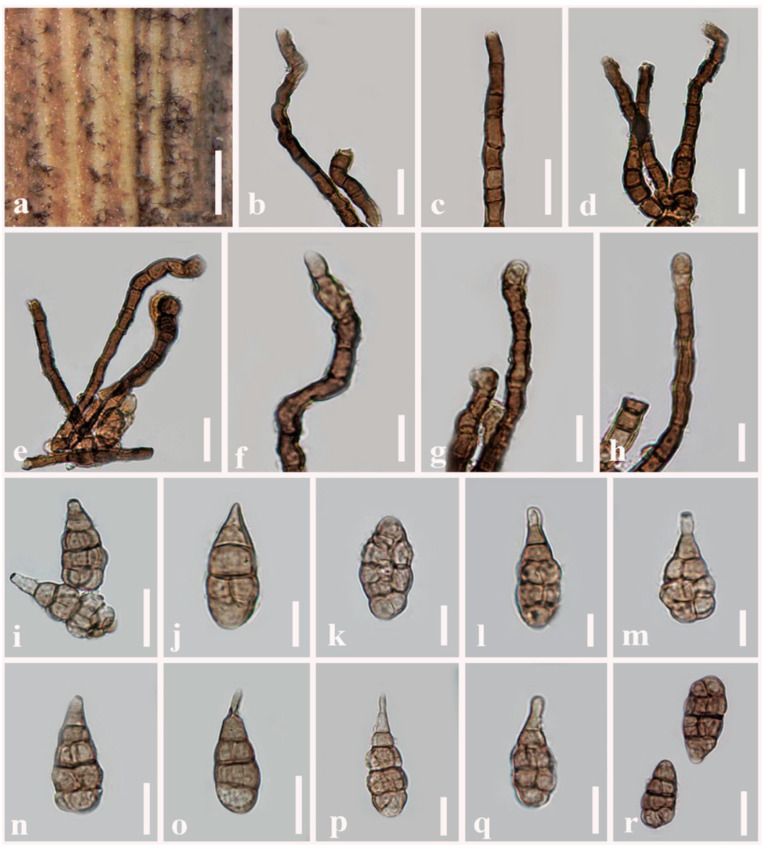
***Alternaria obpyriconidia*** (MFLU 21-0300, holotype). (**a**) Colonies on dead stems of *Vicia faba*; (**b**–**d**) Conidiophores; (**e**–**h**) Conidiophores bearing conidiogenous cells; (**i**–**r**) Conidia. Scale bars: (**a**) = 1000 µm, (**e**) = 50 µm, (**b**–**d**,**f**–**h**,**n**–**q**) = 30 µm, (**i**–**m**,**r**) = 20 µm.

***Alternaria obpyriconidia*** J.F. Li, Camporesi, Phookamsak & Bhat, sp. nov. Figure 3

Index Fungorum number: IF 559797
Etymology: Named after its obpyriform conidia.
Holotype: MFLU 21-0300

Saprobic on dead stems of *Vicia faba* (*Fabaceae*). Sexual morph: Undetermined. Asexual morph: Mycelium superficial on the substrate, composed of septate, branched, smooth, thin-walled, subhyaline to pale white hyphae. Conidiophores (130–)139.5–155 × 11.5–13 µm ($\overset{-}{x}$ = 145.8 × 12.6 µm, *n* = 100), macronematous, mononematous, straight or flexuous, cylindrical, slightly swollen at the apical cell, copper brown to dark brown, septate, unbranched, smooth and thick-walled. Conidiogenous cells 19–23 × 9–12.5 µm ($\overset{-}{x}$ = 19.7 × 10.8 µm, *n* = 100), polytretic, sympodial, integrated, terminal, determinate or percurrent, cylindrical to doliiform, subhyaline, smooth, thick-walled, apically rounded or doliiform, with 2–4 conidiogenous loci. Conidia (58–)62.5–68(–69) × (12.5–)22.5–28(–30) µm ($\overset{-}{x}$ = 64 × 25.4 µm, *n* = 100) acrogenous, solitary, dry, simple, straight or curved, ellipsoidal to obclavate or obpyriform, with short, narrow, pale brown, aseptate, rostrate at beak, pale brown to greyish brown, three to four transverse eusepta, with 1–2 longitudinal to oblique or Y-shaped septa in the middle cells, constricted at the central septum, borne in chain, verruculose or verrucose and thin-walled. Conidial secession schizolytic. 

Culture characteristics: Conidia germinating on PDA within 12 h and germ tubes produced from all cells. Colonies hairy or cottony, pale to dark brown, reaching 5 cm in 7 days at 25 °C, mycelium superficial, effuse, radially striate, with irregular edge, subhyaline to brown hyphae; conidia not formed *in vitro* within 60 days.

Material examined: Italy, Province of Forlì-Cesena, Bagno di Romagna, Valgianna, on dead aerial stems of *Vicia faba* (*Fabaceae*), 29 January 2014, E. Camporesi, IT1688 (MFLU 21-0300, holotype), ex-type living culture = MFLUCC 21-0121; *ibid*., MFLUCC 14-0435.

Notes: In the multi-locus phylogenetic analyses, two strains of *Alternaria obpyriconidia* formed a separate branch basal to *A. macroconidia* (MFLUCC 21-0134), *A. arctoseptata* (MFLUCC 21-0139)*, A. ovoidea* (MFLUCC 14-0427) and *A. baoshanensis* (MFLUCC 21-0124), and also clustered with *A. falcata* (MFLUCC 21-0123). *Alternaria obpyriconidia* differs from *A. macroconidia* in having smaller (58–69 × 12.5–30 µm vs. 68.5–95.5 × 20–30.5), pale brown to greyish brown conidia, with 3–4 transverse eusepta, while *A. macroconidia* has olivaceous brown to golden brown or brown conidia, with 3–5 transverse disto- or eusepta and conidia that are not constricted in *A. macroconidia* [2]. *Alternaria arctoseptata* is distinct from *A. obpyriconidia* in having larger (15–75 × 10–35 µm), yellowish-brown to dark brown, sectored conidia, varied in shape, with 2–3(–6) transverse septa. Conidiophores of *A. arctoseptata* are shorter (50–100 × 8–12 µm vs. (130–)139.5–155 × 11.5–13 µm) and pale brown to light brown, arising from a stomatic base [2], while *A. obpyriconidia* has copper brown to dark brown conidiophores. *Alternaria ovoidea* can be distinguished from *A. obpyriconidia* in having slightly smaller (48–65 × 15.5–30 µm), ovoid, orangish brown to copper brown, sectored, non-beak conidia with 1–3 indistinct transverse septa, whereas *A. obpyriconidia* has short, narrow, pale brown, aseptate, rostrate beak conidia. *Alternaria baoshanensis* can be distinguished from *A. obpyriconidia* in having versicolorous, light brown to dark brown conidiophores, which sometimes branch with several aggregated at the base, and light brown to yellowish brown 3–6 transverse septa conidia [2], whereas *A. obpyriconidia* has unbranched conidiophores. *Alternaria falcata* differs from *A. obpyriconidia* in having smaller (20–50 × 12–23 µm), olivaceous-brown to brown conidia, with 2–5 transverse disto- or eusepta [2]. A nucleotide base comparison of these species is shown in Table 2.

**Figure 4:**
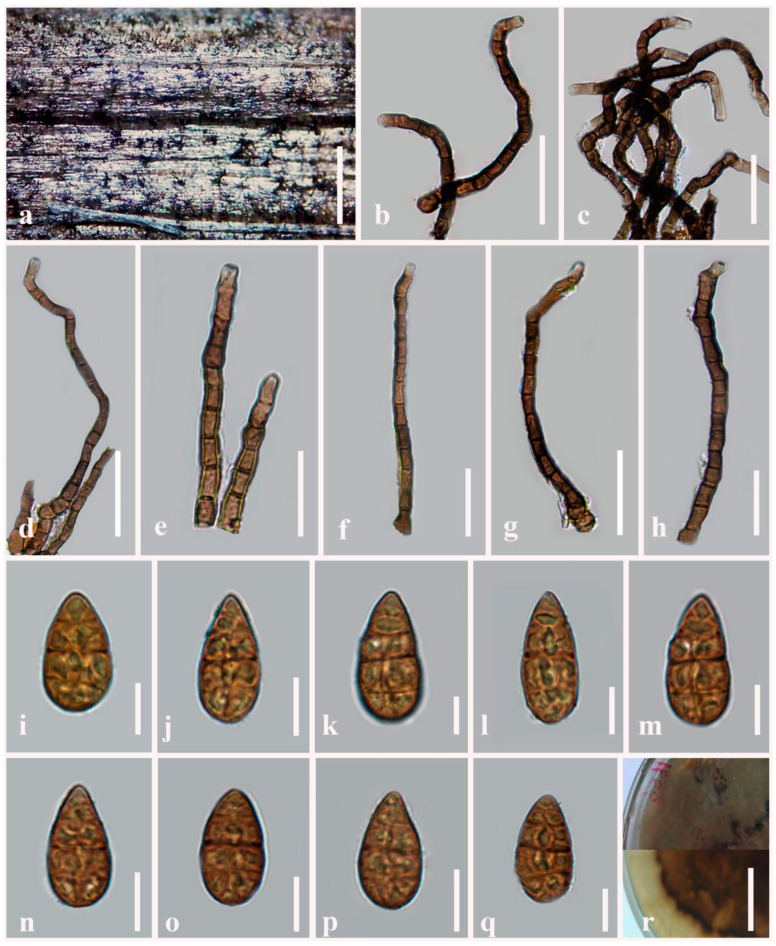
***Alternaria ovoidea*** (MFLU 21-0298, holotype). (**a**) Colonies on dead stem of *Dactylis glomerata*; (**b**–**h**) Conidiophores bearing conidiogenous cells; (**i**–**q**) Conidia; (**r**) Culture on PDA. Scale bars: (**r**) = 2 cm, (**a**) = 100 µm, (**b**–**d**,g) = 50 µm, (**e**,**f**,**h**) = 30 µm, (**i**–**q**) = 20 µm.

***Alternaria ovoidea*** J.F. Li, Camporesi, Bhat & Phookamsak, sp. nov. Figure 4

Index Fungorum number: IF 559798
Etymology: Referring to its ovoid (droplets-like) conidia.
Holotype: MFLU 21-0298

Saprobic on stems of *Dactylis glomerata* (*Poaceae*). Sexual morph: Undetermined. Asexual morph: Mycelium partly superficial on host substrate, composed of septate, branched, smooth, thin-walled, pale brown hyphae. Conidiophores 270–300 × 6.5–11 µm ($\overset{-}{x}$ = 280 × 8 µm, *n* = 100), macronematous, mononematous, copper brown to dark brown, erect, flexuous or sigmoid, cylindrical, septate, branched, smooth to verrucose, thick-walled. Conidiogenous cells 9–13 × 8.5–15 µm ($\overset{-}{x}$ = 9.7 × 11.4 µm, *n* = 100), mono- to polytretic, integrated, terminal, determinate or percurrent, subcylindrical, pale brown to light brown, smooth, thick-walled, apically doliiform with conidiogenous loci cicatrized on conidial secession. Conidia 48–65 × 15.5–30 µm ($\overset{-}{x}$ = 55.4 × 27.2 µm, *n* = 100) acrogenous, solitary, ovoid, orangish brown to copper brown, sectored, with 1–3 indistinct transverse septa, and one longitudinal or oblique or Y-shaped septum in transverse divisions, verruculose, thick-walled. Conidial secession schizolytic.

Culture characteristics: Conidia germinating on PDA within 14 h and germ tubes produced from all cells. Colonies cottony, brown to dark brown, reaching 5 cm in 7 days at 25 °C, mycelium superficial, effuse, radially striated, with irregular edge; conidia not formed *in vitro* within 60 days.

Material examined: Italy, Province of Forlì-Cesena, Fiumicello di Premilcuore, on dead aerial stems of *Dactylis glomerata* (*Poaceae*), 19 January 2014, E. Camporesi, IT1656 (MFLU 21-0298, holotype), ex-type living culture = MFLUCC 14-0427.

Notes: Multi-locus phylogenetic analyses showed that *Alternaria ovoidea* is sister to *A. baoshanensis* with significant support (70% ML, 0.95 PP; Figure 1). *Alternaria ovoidea* differs from *A. baoshanensis* in having solitary, flexuous or sigmoid, copper brown to dark brown conidiophores with a non-stomatic base, while the conidiophores are versicolorous, light brown to dark brown, arising from a stomatic base in *A. baoshanensis*. Conidia of *A. ovoidea* are slightly larger (48–65 × 15.5–30 µm vs. 25–60 × 12–22 µm), orangish brown to copper brown, sectored, with 1–3 indistinct transverse septa, while *A. baoshanensis* has light brown to yellowish brown, sometimes with a short beak, varied in shape, usually subglobose to ellipsoidal, or subcylindrical to obpyriform, 3–6 transverse septa conidia [2]. A nucleotide base comparison of *A. ovoidea* with *A. baoshanensis* showed that they are different in 4/515 bp (0.8%) of ITS, 11/474 bp (2.3%) of *Alt-a1*, 11/567 bp (1.9%) of *gapdh*, 37/559 bp (6.6%) of *rpb2* and 3/238 bp (1.3%) of *tef1-α*.

**Figure 5:**
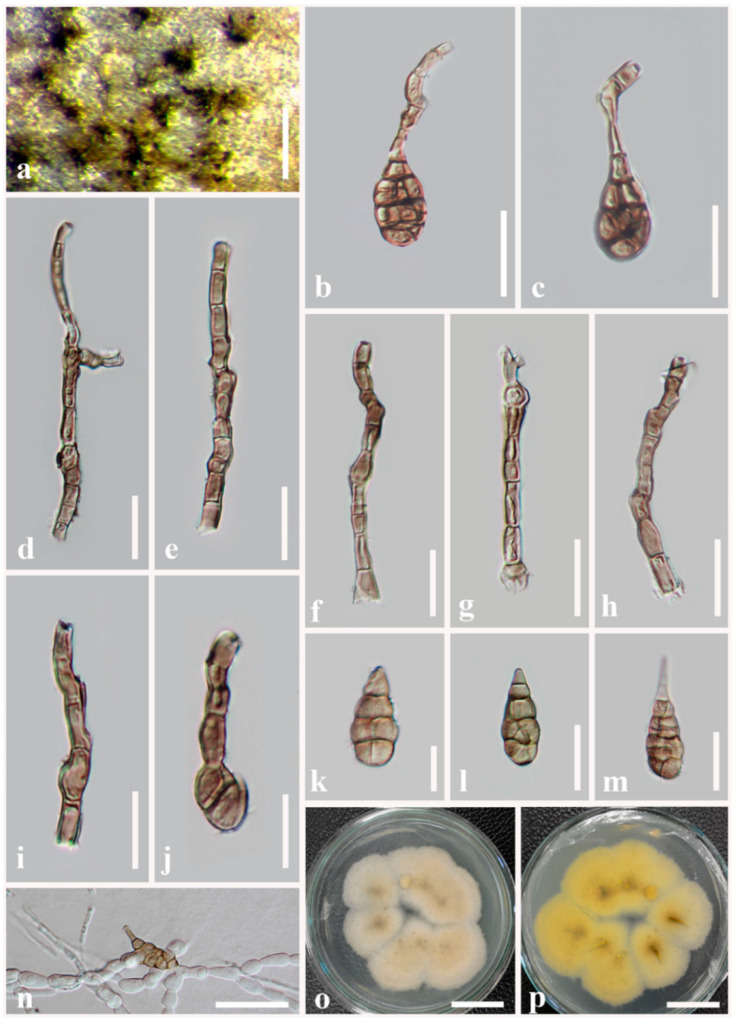
***Alternaria pseudoinfectoria*** (MFLU 21-0311, holotype). (**a**) Colonies on dead stem of *Chenopodium* sp.; (**b**,**c**,**j**) Conidia formed apical secondary conidiophores; (**d**–**i**) Conidiophores; (**k**–**m**) Conidia; (**n**) Germinated conidium; (**o**,**p**) Colonies on PDA. Scale bars: (**o**,**p**) = 2 cm, (**a**) = 300 µm, (**b**–**f**,**h**,**n**) = 20 µm, (**g**,**i**,**j**) = 15 µm, (**k**–**m**) = 10 µm.

***Alternaria pseudoinfectoria*** J.F. Li, Camporesi, Bhat & Phookamsak, sp. nov. Figure 5

Index Fungorum number: IF 559799
Etymology: Referring to the conidial structures resemble *Alternaria* section *infectoriae*.
Holotype: MFLU 21-0311

Saprobic on stems of *Chenopodium* sp. (*Chenopodiaceae*). Sexual morph: Undetermined. Asexual morph: Mycelium superficial on host substrate, composed of septate, branched, smooth, thin-walled, brown hyphae. Conidiophores 55–68 × 12–14 µm ($\overset{-}{x}$ = 62 × 13 µm, *n* = 30), macronematous, mononematous, straight or flexuous, cylindrical, light brown to brown, septate, branched, smooth, thick-walled. Conidiogenous cells 11–12 × 10–14 µm ($\overset{-}{x}$ = 11.5 × 12 µm, *n* = 20), monotretic, integrated, terminal, determinate or percurrent, cylindrical, subhyaline to light brown, smooth, thin-walled, apically doliiform with one conidiogenous locus. Conidia 25–40 × 13–25 µm ($\overset{-}{x}$ = 33 × 19 µm, *n* = 30) acrogenous, holoblastic, solitary, straight, subglobose to obclavate, or obpyriform, sometimes with short, narrow, rostrate, paler brown, septate beak, light brown 3–4 transverse eusepta, with one longitudinal or oblique or Y-shaped septum in some transverse divisions, borne in chain, smooth to minutely verrucose, thin-walled, formed apically secondary conidiophores, with one conidiogenous locus. Conidial secession schizolytic. 

Culture characteristics: Conidia germinating on PDA within 14 h and germ tubes produced from all cells. Colonies immersed in PDA, cottony, white to grey, reaching 5 cm in 7 days at 25 °C, mycelium superficial, effuse, radially striate, with irregular edge, white hyphae; conidia not sporulated *in vitro* within 60 days.

Material examined: Italy, Province of Forlì-Cesena, Forlì, Via Nenni, on dead aerial stems of *Chenopodium* sp. (*Chenopodiaceae*), 17 October 2014, E. Camporesi, IT2181 (MFLU 21-0311, holotype), ex-type living culture = MFLUCC 21-0126.

Notes: *Alternaria pseudoinfectoria* resembles species in sect. *Infectoriae* due to its conidia often developing long secondary conidiophores. Although species in section *Panax* also formed long secondary conidiophores, conidiogenous loci on secondary conidiophores are rather monotretic in *A. pseudoinfectoria*, which more resemble structures of species in sect. *Infectoriae* [14,47]. However, *A. pseudoinfectoria* corresponds with sect. *Alternaria* in having straight or curved primary conidiophores, simple to branched, with one apical conidiogenous locus, and conidia born in chain [8]. In phylogenetic analyses, two strains of *A. pseudoinfectoria* formed a well-resolved subclade (82% ML, 0.99 PP) and is sister to *A. muriformispora* with 76% ML and 0.98 PP support (Figure 1). The morphological comparison of these two species is detailed in notes of *A. muriformispora*.

**Figure 6:**
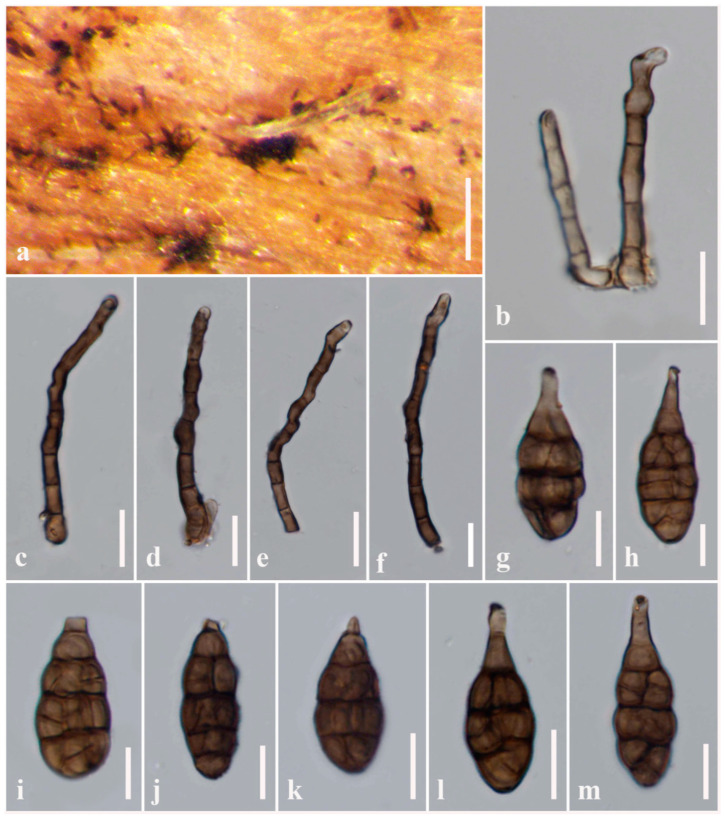
***Alternaria rostroconidia*** (MFLU 21-0318, holotype). (**a**) Colonies on dead stems of *Arabis* sp; (**b**–**f**) Conidiophores bearing conidiogenous cells (**g**–**m**) Conidia. Scale bars: (**a**) = 200 µm, (**e**) = 50 µm, (**b**,**d**,**f**) = 30 µm, (**c**,**g**–**m**) = 20 µm.

***Alternaria rostroconidia*** J.F. Li, Camporesi, Bhat & Phookamsak, sp. nov. Figure 6

Index Fungorum number: IF 559800
Etymology: Referring to the rostrate conidia.
Holotype: MFLU 21-0318

Saprobic on dead stems of *Arabis* sp. (*Brassicaceae*). Sexual morph: Undetermined. Asexual morph: Mycelium superficial on host substrate, with dark hyphae. Conidiophores 105–120 × 11–15 µm ($\overset{-}{x}$ = 112 × 13 µm, *n* = 30), macronematous, solitary or 2–5 aggregated at the base, straight or flexuous, cylindrical, light brown to dark brown, septate, geniculate, smooth or sometimes semi-verrucose, thick-walled. Conidiogenous cells 12–18 × 5–8 µm ($\overset{-}{x}$ = 15 × 6 µm, *n* = 20), mono- to polytretic, normally sympodial proliferations, integrated, terminal, determinate or percurrent, cylindrical, subhyaline or semi-colored, smooth, thin-walled, apically doliiform, with 1–2 conidiogenous loci and swollen knots near conidiogenous loci. Conidia 50–80 × 25–30 µm ($\overset{-}{x}$ = 66 × 22 µm, *n* = 30) acrogenous, solitary, straight or curved, ellipsoidal or ovoid to obpyriform, with short, narrow, rostrate, paler brown, aseptate beak, with distinct hilum at the apex, dark brown, 3–4 transverse eusepta, with one longitudinal or oblique or Y-shaped septum in some transverse divisions, sometimes sectored, slightly constricted at the septa, borne in chain, smooth, thick-walled. Conidial secession schizolytic.

Culture characteristics: Conidia germinating on PDA within 12 h and germ tubes produced from lateral cells. Colonies cottony, brown to dark brown, reaching 5 cm in 10 days at 25 °C, mycelium superficial, effuse, radially striate, with irregular edge, white to grey hyphae; conidia not sporulated *in vitro* within 60 days.

Material examined: Italy, Province of Forlì-Cesena, Premilcuore, on dead aerial stems of *Arabis* sp. (*Brassicaceae*), 8 October 2017, E. Camporesi, IT3515 (MFLU 21-0318, holotype), ex-type living culture, MFLUCC 21-0136.

Notes: *Alternaria rostroconidia* corresponds with species in sect. *Alternaria* in having obpyriform, born in chain conidia with several transverse and longitudinal septa [8]. In multi-locus phylogenetic analyses, *A. rostroconidia* has a close relationship with *A. minimispora* with significant support in BI analyses (0.96 PP; Figure 1). A *rpb2* nucleotide pairwise comparison showed that *A. rostroconidia* differs from *A. minimispora* in 19/505 bp (3.8% difference, no gap). In *gapdh*, *A. rostroconidia* differs from *A. minimispora* in 10/545 bp (1.8% difference, no gap). The *Alt-a1* nucleotide pairwise comparison shows that *A. rostroconidia* differs from *A. minimispora* in 8/474 bp (1.7% difference, no gap). Morphologically, *A. rostroconidia* can be distinguished from *A. minimispora* in having larger (50–80 × 25–30 µm vs. 13–25 × 8–11 µm), ellipsoidal or ovoid to obpyriform conidia, with 3–4 transverse eusepta and short, narrow, rostrate and distinct hilum at the apex. *Alternaria minimispora* has subglobose to ovoid, sometimes obpyriform or obturbinate, beakless, two to four transversely euseptate conidia [2].

**Figure 7:**
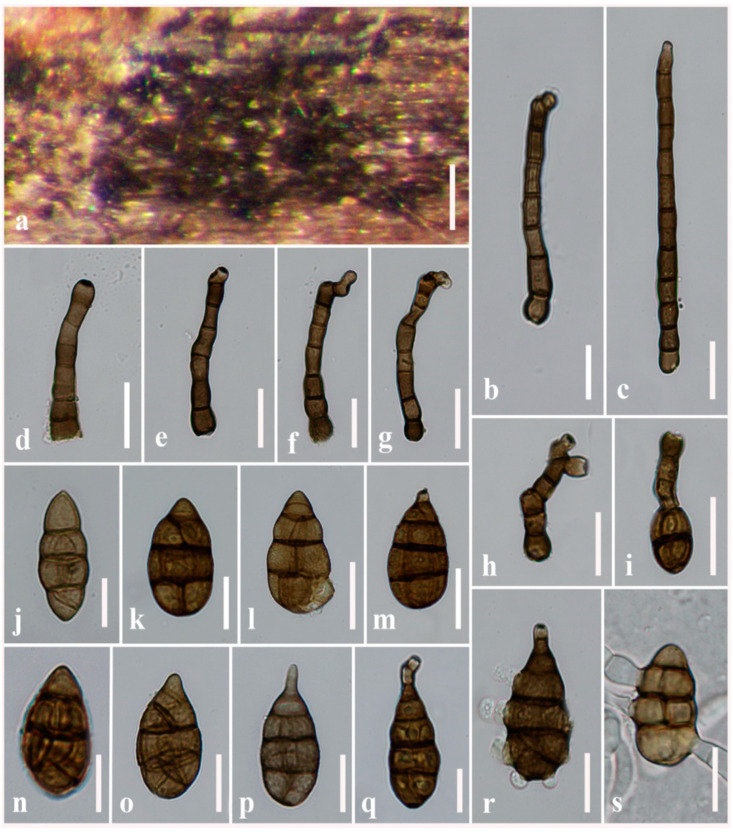
***Alternaria torilis*** (MFLU 21-0299, holotype). (**a**) Colonies on stems of *Torilis arvensis*; (**b**–**h**) Conidiophores bearing conidiogenous cells; (**i**) Secondary conidiophores arising from conidium; (**j**–**q**) Conidia; (**r**,**s**) Germinated conidia. Scale bars: (**a**) = 200 µm, (**c**,**g**) = 30 µm, (**b**,**d**–**f**,**h**–**s**) = 20 µm.

***Alternaria torilis*** J.F. Li, Camporesi, Bhat & Phookamsak, sp. nov. Figure 7

Index Fungorum number: IF 559801
Etymology: Named after the host genus “*Torilis*”.
Holotype: MFLU 21-0299

Saprobic on dead aerial stems of *Torilis arvensis* (*Apiaceae*). Sexual morph: Undetermined. Asexual morph: Mycelium superficial on host substrate, composed of septate, branched, smooth, thin-walled, brown to light brown hyphae. Conidiophores (155–)177–185(–191) × (7.5–)8–10(–11) µm ($\overset{-}{x}$ = 175.2 × 8.8 µm, *n* = 100), macronematous, mononematous, straight or flexuous, cylindrical, dark brown, unbranched, septate, sometimes branched, smooth, thick-walled. Conidiogenous cells 7–9(–10) × (6.5–)7.5–10 µm ($\overset{-}{x}$ = 8.2 × 8.9 µm, *n* = 100), mono- to polytretic, integrated, terminal, determinate or percurrent, cylindrical, subhyaline, smooth, thin-walled, apically doliiform, with 2 conidiogenous loci cicatrized on conidial secession. Conidia (55–)60–75(–82) × (23–)25–31.5(–32) µm ($\overset{-}{x}$ = 68.5 × 28.5 µm, *n* = 100) acrogenous, solitary, dry, straight, fusiform to ovoid, or obturbinate to obpyriform, sometimes with short, narrow, pale brown to light brown, aseptate beak, brown to dark brown, 2–4 transverse eusepta, with one longitudinal or oblique or Y-shaped distoseptum in some transverse divisions, borne in chain, minutely verruculose, thin-walled, formed apically secondary conidiophores with one conidiogenous locus. Conidial secession schizolytic.

Culture characteristics: Conidia germinating on PDA within 14 h and germ tubes produced from lateral cells. Colonies growing on PDA, hairy or cottony, light brown to brown, reaching 5 cm in 14 days at 25 °C, mycelium superficial, effuse, radially striate, with irregular edge, colorless hyphae. Conidia sporulated on OA within 15 days, phragmosporous to muriform, oblong to ovoid, brown to dark brown, with short, doliiform, apical beak, formed apically or laterally, short, branched or unbranched secondary conidiophores with one to two conidiogenous loci at apex and 1–3 transverse septa, with 1–2 longtudinal or Y-shape septa in transverse division, smooth to minutely verrucose and thin-walled.

Material examined: Italy, Province of Forlì-Cesena, Forlì, San Lorenzo in Noceto, on dead aerial stems of *Torilis arvensis* (*Apiaceae*), 23 January 2014, E. Camporesi, IT1667 (MFLU 21-0299, holotype), ex-type living culture = MFLUCC 14-0433, *ibid*., MFLUCC 21-0133.

Notes: *Alternaria torilis* resembles *A. alternata* in having a brown to dark brown short beak, 2–4 transverse septa conidia and forming secondary conidiophores. The conidial body can narrow gradually into a tapered beak or secondary conidiophore, with curved primary conidiophores and solitary conidiophores with mono- to polytretic conidiophores with conidiogenous loci at the apex. *Alternaria torilis* differs from *A. alternata* by its darker, ovoid to obturbinate or obpyriform, which is rather ovoid to chiefly obclavate or obpyriform in *A. alternata*. Conidiophores of *A. torilis* normally have 2 conidiogenous loci and are rostrate at the apex. In the phylogenetic analyses, three strains of *A. torilis* formed a well-resolved subclade (85% Ml, 1.00 PP; Figure 1), independently constituted within sect. *Alternaria*, and have a close relationship with *A. ellipsoidialis* and *A. eupatoriicola* distancing from *A. alternata*. *Alternaria torilis* can be distinguished from *A. ellipsoidialis* in having larger (55–82 × 23–32 µm vs. 35–60 × 18–25 µm), fusiform to ovoid, or obturbinate to obpyriform, brown to dark brown conidia, with 2–4 transverse eusepta. *Alternaria ellipsoidialis* has oblong to ellipsoidal, or ovoid, pale brown to brown, sectored, 4–7 transverse eusepta conidia [2]. *Alternaria eupatoriicola* is different from *A. torilis* in having smaller (40–65 × 15–30 µm vs. 55–82 × 23–32), ovoid to obpyriform, reddish brown to brown, 3–5transverse septa conidia. In addition, conidia of *A. torilis* formed apically secondary conidiophores with one conidiogenous locus, whereas it was absent in *A. eupatoriicola* [2].

The nucleotide pairwise comparison of the ITS showed that *Alternaria* *torilis* differs from *A. alternata* (CBS 916.96, ex-type) in 9/485 bp (1.9% difference, no gap), differs from *A. ellipsoidialis* in 10/485 bp (2.1% difference, no gap) and differs from *A. eupatoriicola* in 9/480 bp (1.9% difference, no gap). A *rpb2* nucleotide pairwise comparison showed that *A. torilis* differs from *A. alternata* (CBS 916.96, ex-type) in 42/558 bp (7.5% difference, no gap), differs from *A. ellipsoidialis* in 9/480 bp (1.9% difference, no gap) and differs from *A. eupatoriicola* in 40/558 bp (7.2% difference, no gap). A *gapdh* nucleotide pairwise comparison showed that *A. torilis* differs from *A. alternata* (CBS 916.96, ex-type) in 31/590 bp (5.3% difference, no gap), differs from *A. ellipsoidialis* in 18/560 bp (3.2% difference, no gap) and differs from *A. eupatoriicola* in 25/590 bp (4.2% difference, no gap). The nucleotide pairwise comparison of the *Alt-a1* showed that *A. torilis* differs from *A. alternata* (CBS 916.96, ex-type) in 25/465 bp (5.4% difference, no gap), differs from *A. ellipsoidialis* in 20/465 bp (4.3% difference, no gap) and differs from *A. eupatoriicola* in 15/470 bp (3.2% difference, no gap).

## 4. Discussion and Conclusions

The aim of the present study was to introduce six novel *Alternaria* species in sect. *Alternaria* based on a morpho-molecular approach. These six saprobic species occurred on a variety of host plants in families *Apiaceae*, *Brassicaceae*, *Chenopodiaceae*, *Fabaceae*, *Plantaginaceae*, and *Poaceae* in Italy and could not be ascribed to any known taxa within sect. *Alternaria*. According to a recent classification provided by Woudenberg et al. [17] and Gannibal [15], we also note the morphological differences among extant species in this section. Hence, six new species: *A. muriformispora*, *A. obpyriconidia*, *A. ovoidea*, *A. pseudoinfectoria*, *A. rostroconidia* and *A. torilis* are introduced, described and illustrated herein.

Multi-locus phylogeny, based on a concatenated ITS, LSU, SSU, *tef1-α*, *rpb2*, *gapdh* and *Alt-a1* DNA sequence matrix, revealed that these novel species formed well-resolved subclades within the sect. *Alternaria,* except for *A. obpyriconidia* that formed a distinct branch with other closely related species with low support in ML, but well-resolved species in BI analysis (1.00 PP; Figure 1). Based on the phylogenetic analyses and morphological characteristics, coupled with host preferences and nucleotide polymorphisms, *A. obpyriconidia* is justified as a new species following Jeewon and Hyde [48]. Furthermore, these six new species are distant from *A. arborescens* species complex (AASC) and *A. alternata* as well as other species in this section, which provided further evidence to support their phylogenetic affinities within the sect. *Alternaria*. 

In the present analyses, *Alternaria doliconidium* and *A. italica* formed subclades, constituted within *A. alternata*, and that concurred with Li et al. [2]. Even though Woudenberg et al. [17] accepted only 11 phylogenetic species and one species complex in sect. *Alternaria*, and also treated 35 morphospecies as synonyms of *A. alternata*, Li et al. [2] re-analyzed the isolates of *A. alternata* with their new collections and mentioned that *A. alternata* could be separated to be at least five distinct species. However, more evidence is needed to support this conclusion. Similarly, *A. doliconidium* and *A. italica* lack informative cording genes such as *Alt-a1*, *gapdh*, *rpb2* and *tef1-α* to justify their heterospecific status, with *A. alternata* pending further studies.

Woudenberg et al. [17] indicated that *Alternaria* species, including *Alternaria* sect. *Alternaria*, should be delineated by using phylogenomics due to a lack of effective gene sequences; however, the multi-locus phylogenetic analyses could well delineate species in sect. *Alternaria* (Figure 1) in studies of Wanasinghe et al. [20], Jayawardena et al. [21], Nishikawa and Nakashima [22] and Li et al. [2]. In the present study, phylogenetically analyzed taxa in sect. *Alternaria*, based on combined the intervening ITS regions, nuclear ribosomal DNA SSU, LSU and protein-coding genes *Alt-a1*, *tef1-α*, *gapdh* and *rpb2*, demonstrated that the recent taxa in this section formed distinct clades and were well supported in the phylogenetic tree. Nucleotide polymorphic comparisons also show the differences between our new taxa, which support the justifications of the new species described herein. It is interesting to note that in the nucleotide polymorphic comparisons of gene sequences among the species in *Alternaria* sect. *Alternaria*, *rpb2* contains the most nucleotide differences among the species (up to 3.5%), which implies that this protein-cording gene may be a potentially effective gene region to delineate species in sect. *Alternaria*. 

Nevertheless, species of *Alternaria* in sect. *Alternaria* are similar in morphological characteristics, and it is difficult to distinguish these species based solely on morphology. However, the conidial characteristics (e.g., conidial septation and rostrate or non-beak conidia) of our six novel species are significant to distinguish them from other species. Multi-locus phylogenetic analyses also provided further evidence, confirming that these six species are novel. These six species clearly formed a separate branch with significant support values (≥70% ML and 0.95 PP; Figure 1) in the present study, and this concurs with the findings of Li et al. [2]. Jeewon and Hyde [48] suggested that the nucleotide polymorphic comparisons of reliable genes should be more than 1.5% different for justifying the novel species. Even though the ITS, LSU, SSU and *tef1-α* could not be used to delineate some species in sect. *Alternaria*, the remaining gene regions (i.e., *Alt-a1*, *gapdh* and *rpb2*) proved sufficient for distinguishing these new species. Therefore, the novel species introduced herein were justified based on the multi-locus phylogeny coupled with morphological characteristics and nucleotide polymorphic comparisons of reliable genes.

## Figures and Tables

**Table 1:** Taxa used for the phylogenetic analyses in this study and their GenBank accession numbers. The ex-type cultures are indicated with superscript “^T^” and the newly generated sequences are indicated in bold.

Species Name	Strains/Voucher No.	GenBank Accession Numbers						
		SSU	LSU	*rpb2*	ITS	*gapdh*	*tef1-α*	*Alt-a1*
*Alternaria alstroemeriae*	CBS 118808	KP124917	KP124447	KP124764	KP124296	KP124153	KP125071	KP123845
*Alternaria alstroemeriae*	CBS 118809 ^T^	NG063029	NG069882	KP124765	NR163686	KP124154	KP125072	MH084526
*Alternaria alternantherae*	CBS 124392	KC584506	KC584251	KC584374	KC584179	KC584096	KC584633	KP123846
*Alternaria alternata*	CBS 102596	KP124950	MH874392	KP124796	MH862796	KP124183	KP125104	KP123877
*Alternaria alternata*	CBS 102599	KP124952	MH874395	KP124798	MH862799	KP124185	KP125106	KP123879
*Alternaria alternata*	CBS 102600	KP124953	MH874396	KP124799	MH862800	KP124186	KP125107	KP123880
*Alternaria alternata*	CBS 102602	KP124954	MH877754	KP124800	KP124332	KP124187	KP125108	KP123881
*Alternaria alternata*	CBS 102603	KP124955	KP124485	KP124801	KP124333	KP124188	KP125109	KP123882
*Alternaria alternata*	CBS 102604	KP124956	MH874399	KP124802	MH862803	-	KP125110	-
*Alternaria alternata*	CBS 113013	KP124963	KP124493	KP124809	KP124341	KP124195	KP125117	KP123889
*Alternaria alternata*	CBS 113014	KP124964	KP124494	KP124810	KP124342	KP124196	KP125118	KP123890
*Alternaria alternata*	CBS 113015	KP124965	KP124495	KP124811	KP124343	KP124197	KP125119	KP123891
*Alternaria alternata*	CBS 119399	KP124983	KP124513	KP124829	KP124361	-	KP125137	KP123910
*Alternaria alternata*	CBS 120829	KP124986	KP124516	KP124832	KP124364	KP124216	KP125140	KP123912
*Alternaria alternata*	CBS 121344	KP124988	KP124518	KP124834	KP124365	KP124217	KP125142	KP123913
*Alternaria alternata*	CBS 121346	KP124989	KP124519	KP124835	KP124366	KP124218	KP125143	KP123914
*Alternaria alternata*	CBS 121492	KP124994	KP124524	KP124840	KP124370	KP124222	KP125148	KP123918
*Alternaria alternata*	CBS 124277	KP124997	KP124527	KP124843	KP124373	KP124225	KP125151	KP123921
*Alternaria alternata*	CBS 124278	KP124998	KP124528	KP124844	KP124374	KP124226	KP125152	KP123922
*Alternaria alternata*	CBS 916.96 ^T^	KC584507	DQ678082	KC584375	AF347031	AY278808	KC584634	-
*Alternaria arborescens*	CBS 101.13	KP125016	KP124546	KP124862	KP124392	KP125170	KP124244	KP123940
*Alternaria arborescens*	CBS 105.24	KP125017	KP12454	KP124863	KP124393	KP125171	KP124245	KP123941
*Alternaria arborescens*	CBS 105.49	KP125020	KP124550	KP124866	KP124396	KP125174	KP124248	KP123944
*Alternaria arborescens*	CBS 108.41	KP125018	KP124548	KP124864	KP124394	KP125172	KP124246	KP123942
*Alternaria arborescens*	CBS 113.41	KP125019	KP124549	KP124865	KP124395	KP125173	KP124247	KP123943
*Alternaria arborescens*	CBS 750.68	KP125021	KP124551	KP124868	KP124398	KP125176	KP124250	KP123945
*Alternaria arborescens*	CBS 102605 ^T^	KC584509	KC584253	KC584377	AF347033	AY278810	KC584636	AY563303
*Alternaria arborescens*	CBS 109730	KP125022	KP124552	KP124869	KP124399	KP125177	KP124251	KP123946
*Alternaria arborescens*	CBS 119544	NG063030	NG_069254	KP124878	MH863062	KP125186	JQ646321	KP123955
*Alternaria arborescens*	CBS 119545	KP125032	KP124562	KY392798	KP124409	KP125187	KP124260	KP123956
*Alternaria arborescens*	CBS 123267	KP125035	KP124565	KP124882	KP124412	KP125190	KP124263	KP123959
*Alternaria arctoseptata*	MFLUCC 21-0139 ^T^	MZ621874	MZ621948	OK236655	-	0K236608	OK236702	OK236755
*Alternaria betae-kenyensis*	CBS 118810 ^T^	NG_063032	NG_069256	JQ905180	NR136118	JQ905161	KP125197	JQ905104
*Alternaria baoshanensis*	MFLUCC 21-0124 ^T^	MZ621878	MZ621952	OK236659	MZ622003	OK236613	OK236706	OK236760
*Alternaria breviconidiophora*	MFLUCC 22-0075 ^T^	MZ621870	MZ621944	OK236651	MZ621997	OK236604	OK236698	OK236751
*Alternaria breviconidiophora*	MFLU 21-0317	MZ621871	MZ621945	OK236652	MZ621998	OK236605	OK236699	OK236752
*Alternaria burnsii*	CBS 107.38 ^T^	NG063033	N069257	JQ646457	NR136119	JQ646305	KP125198	JQ646388
*Alternaria burnsii*	CBS 110.50	KP125044	KP124574	KP124890	KP124421	KP124271	KP125199	KP123968
*Alternaria burnsii*	CBS 118816	KP125046	KP124576	KP124892	KP124423	KP124273	KP125201	KP123970
*Alternaria burnsii*	CBS 118817	KP125047	KP124577	KP124893	KP124424	KP124274	KP125202	KP123971
*Alternaria burnsii*	CBS 130264	KP125048	KP124578	KP124894	KP124425	KP124275	KP125203	KP123972
*Alternaria doliconidium*	KUN-HKAS 100840^T^	NG065142	NG069551	-	NR158361	-	-	-
*Alternaria doliconidium*	KUMCC 17-0263 ^T^	MG829094	MG828980	-	MG828864	-	-	-
*Alternaria eichhorniae*	CBS 119778	KP125050	KP124580	KP124896	KP124426	KP124277	KP125205	-
*Alternaria eichhorniae*	CBS 489.92 ^T^	NG063034	KP124579	KP124895	-	KP124276	KP125204	KP123973
*Alternaria ellipsoidialis*	MFLUCC 21-0132 ^T^	MZ621862	MZ621936	OK236643	MZ621989	OK236596	OK236690	OK236743
*Alternaria eupatoriicola*	MFLUCC 21-0122 ^T^	MZ621855	MZ621929	OK236636	MZ621982	OK236589	OK236683	OK236736
*Alternaria falcata*	MFLUCC 21-0123 ^T^	MZ621865	MZ62139	OK236649	MZ621992	OK236599	OK236693	OK236746
*Alternaria gaisen*	CBS 118488 ^T^	KP125051	KP124581	KP124897	KP124427	KP124278	KP125206	KP123975
*Alternaria gaisen*	CBS 632.93	KC584531	KC584275	KC584399	KC584197	KC584116	KC584658	KP123974
*Alternaria gaisen*	CPC 25268	KP125052	KP124582	KP124898	KP124428	KP124279	KP125207	KP123976
*Alternaria gossypina*	CBS 100.23	KP125053	KP124583	KP124899	KP124429	KP124280	KP125208	KP123977
*Alternaria gossypina*	CBS 104.32 ^T^	KP125054	KP124584	KP124900	KP124430	JQ646312	KP125209	JQ646395
*Alternaria gossypina*	CBS 107.36	KP125055	KP124585	KP124901	KP124431	-	KP125210	-
*Alternaria gossypina*	CBS 102597	KP125056	MH874393	KP124902	MH862797	KP124281	KP125211	KP123978
*Alternaria gossypina*	CBS 102601	KP125057	MH874397	KP124903	MH862801	KP124282	KP125212	KP123979
*Alternaria iridiaustralis*	CBS 118486 ^T^	NG_063035	NG_069258	KP124905	NR_136120	KP124284	KP125214	KP123981
*Alternaria iridiaustralis*	CBS 118487	KP125060	KP124590	KP124906	KP124436	KP124285	KP125215	KP123982
*Alternaria iridiaustralis*	CBS 118404	KP125058	KP124588	KP124904	KP124434	KP124283	KP125213	KP123980
*Alternaria italica*	KUMCC 17-0090	-	-	-	MG764018	-	-	-
*Alternaria italica*	MFLUCC 14-0421 ^T^	-	MG818319	MG859737	MG764017	-	-	-
*Alternaria jacinthicola*	CBS 133751 ^T^	KP125062	KP124592	KP124908	KP124438	KP124287	KP125217	KP123984
*Alternaria jacinthicola*	CBS 878.95	KP125061	KP124591	KP124907	KP124437	KP124286	KP125216	KP123983
*Alternaria jacinthicola*	CPC 25267	KP125063	KP124593	KP124909	KP124439	KP124288	KP125218	KP123985
*Alternaria lathyri*	MFLUCC 21-0140 ^T^	MZ621847	MZ621921	OK236628	MZ621974	OK236581	OK236675	OK236728
*Alternaria lathyri*	MFLU 21-0297	MZ621848	MZ621922	OK236629	MZ621975	OK236582	OK236676	OK236729
*Alternaria longipes*	CBS 113.35	KP125064	KP124594	KP124910	KP124440	KP124289	KP125219	KP123986
*Alternaria longipes*	CBS 121332	KP125067	KP124597	KP124913	KP124443	KP124292	KP125222	KP123989
*Alternaria longipes*	CBS 121333	KP125068	KP124598	KP124914	KP124444	KP124293	KP125223	KP123990
*Alternaria longipes*	CBS 539.94	KP125065	KP124595	KP124911	KP124441	KP124290	KP125220	KP123987
*Alternaria longipes*	CBS 540.94	KC584541	KC584285	KC584409	-	-	KC584667	-
*Alternaria longipes*	CBS 917.96	KP125066	KP124596	KP124912	KP124442	KP124291	KP125221	KP123988
*Alternaria macilenta*	MFLUCC 21-0138 ^T^	MZ621845	MZ621919	OK236626	MZ621972	OK236579	OK236673	OK236726
*Alternaria macroconidia*	MFLUCC 21-0134 ^T^	MZ621876	MZ621950	OK236657	MZ622001	OK236610	OK236704	OK236757
*Alternaria minimispora*	MFLUCC 21-0127 ^T^	MZ621853	MZ621927	OK236634	MZ621980	OK236587	OK236681	OK236734
***Alternaria muriformispora***	**MFLUCC 22-0073 ^T^**	**MZ621849**	**MZ621923**	**OK236630**	**MZ621976**	**OK236583**	**OK236677**	**OK236730**
***Alternaria muriformispora***	**MFLU 21-0309**	**MZ621850**	**MZ621924**	**OK236631**	**MZ621977**	**OK236584**	**OK236678**	**OK236731**
*Alternaria oblongoellipsoidea*	MFLUCC 22-0074 ^T^	MZ621840	MZ621914	OK236621	MZ621967	OK236574	OK236668	OK236721
***Alternaria obpyriconidia***	**MFLUCC 21-0121 ^T^**	**MZ621851**	**MZ621925**	**OK236633**	**MZ621978**	**OK236585**	**OK236680**	**OK236732**
***Alternaria obpyriconidia***	**MFLU 21-0300**	**MZ621852**	**MZ621926**	**OK236632**	**MZ621979**	**OK236586**	**OK236679**	**OK236733**
*Alternaria orobanches*	MFLUCC 21-0137^T^	MZ621882	MZ621956	-	MZ622007	-	OK236710	OK236763
***Alternaria ovoidea***	**MFLUCC 14-0427** **^T^**	**MZ621880**	**MZ621954**	**OK236661**	**MZ622005**	**OK236614**	**OK236708**	**OK236761**
*Alternaria phragmiticola*	MFLUCC 21-0125 ^T^	MZ621867	MZ621941	OK236649	MZ621994	OK236602	OK236696	OK236749
*Alternaria phragmiticola*	MFLU 21-0316	MZ621868	MZ621942	OK236650	MZ621995	OK236603	OK236697	OK236750
***Alternaria pseudoinfectoria***	**MFLUCC 21-0126 ^T^**	**MZ621857**	**MZ621931**	**OK236638**	**MZ621984**	**OK236591**	**OK236685**	**OK236738**
***Alternaria pseudoinfectoria***	**MFLU 21-0311**	**MZ621858**	**MZ621932**	**OK236639**	**MZ621985**	**OK236592**	**OK236686**	**OK236739**
***Alternaria rostroconidia***	**MFLUCC 21-0136 ^T^**	**MZ621842**	**MZ621916**	**OK236623**	**MZ621969**	**OK236576**	**OK236670**	**OK236723**
***Alternaria rostroconidia***	**MFLU 21-0318**	**MZ621843**	**MZ621917**	**OK236624**	**MZ621970**	**OK236577**	**OK236671**	**OK236724**
*Alternaria salicicola*	MFLUCC 22-0072 ^T^	MZ621872	MZ621946	OK236653	MZ621999	OK236606	OK236700	OK236753
*Alternaria* sp.	CBS 108.27	KC584601	KC584343	KC584468	KC584236	KC584162	KC584727	-
*Alternaria tomato*	CBS 103.30	KP125069	KP124599	KP124915	KP124445	KP124294	KP125224	KP123991
*Alternaria tomato*	CBS 114.35	KP125070	KP124600	KP124916	KP124446	KP124295	KP125225	KP123992
***Alternaria torilis***	**MFLUCC 21-0133**	**MZ621859**	**MZ621933**	**OK236640**	**MZ621986**	**OK236593**	**OK236687**	**OK236740**
***Alternaria torilis***	**MFLU 21-0299**	**MZ621860**	**MZ621934**	**OK236642**	**MZ621987**	**OK236595**	**OK236689**	**OK236742**
***Alternaria torilis***	**MFLUCC 14-0433 ^T^**	**MZ621861**	**MZ621935**	**OK236641**	**MZ621988**	**OK236594**	**OK236688**	**OK236741**
*Alternaria vitis*	MFLUCC 17-1109 ^T^	-	-	-	MG764007	-	-	-

Abbreviations: CBS: the Westerdijk Fungal Biodiversity Institute, Utrecht, The Netherlands; CPC: Culture Collection of Pedro Crous, Netherlands; KUMCC: Kunming Institute of Botany Culture Collection, Yunnan, China; KUN-HKAS: Herbarium of Cryptogams Kunming Institute of Botany Academia Sinica, Yunnan, China; MFLU: the Herbarium of Mae Fah Luang University Chiang Rai, Thailand; MFLUCC: Mae Fah Luang University Culture Collection, Chiang Rai, Thailand.

**Table 2:** A nucleotide base comparison of *Alternaria obpyriconidia* with other phylogenetically related species.

Species	Nucleotide Base Difference of Each Informative Gene Regions				
	*Alt-a1*	*gapdh*	ITS	*rpb2*	*tef1-α*
*Alternaria arctoseptata*	11/476 bp (2.3%)	15/570 bp (2.6%)	-	39/560 bp (7.0%)	4/240 bp (1.7%)
*A. baoshanensis*	8/474 bp (1.7%)	15/568 bp (2.6%)	5/515 bp (1%)	40/559 bp (7.2%)	3/240 bp (1.3%)
*A. falcata*	10/474 bp (2.1%)	12/568 bp (2.1%)	5/515 bp (1%)	37/559 bp (6.6%)	4/240 bp (1.7%)
*A. macroconidia*	11/474 bp (2.3%)	11/567 bp (1.9%)	4/515 bp (0.8%)	54/560 bp (9.6%)	4/240 bp (1.7%)
*A. ovoidea*	16/470 bp (3.4%)	14/568 bp (2.5%)	4/515 bp (0.8%)	42/559 bp (7.5%)	3/240 bp (1.3%)

## Data Availability

All data availability was mentioned in the manuscript. The novel taxa were registered in Index Fungorum (http://www.indexfungorum.org/Names/Names.asp, accessed on 15 July 2022) including Index Fungorum numbers IF 559795, IF 559797, IF 559798, IF 559799, IF 559800 and IF 559801. The newly generated sequences were deposited in GenBank (https://www.ncbi.nlm.nih.gov/genbank/submit/, accessed on 25 July 2021) and the GenBank accession numbers were shown in Table 1.
